# Rethinking Normal Thyroid-Stimulating Hormone (TSH): Stroke Risk and Metabolic Implications in High-Risk Cardiovascular Patients

**DOI:** 10.7759/cureus.94854

**Published:** 2025-10-18

**Authors:** Lia Cals, Maria Zanella, Simone Matsuda, Marcelo Batista, Glaucia Carneiro

**Affiliations:** 1 Endocrinology, Diabetes, and Metabolism, Universidade Federal de Medicina de São Paulo, São Paulo, BRA; 2 Endocrinology, Diabetes, and Metabolism, Universidade Federal de Medicina de São Paulo, Sâo Paulo, BRA; 3 Nephrology, Universidade Federal de Medicina de São Paulo, São Paulo, BRA; 4 Endocrinology, Universidade Federal de Medicina de São Paulo, São Paulo, BRA

**Keywords:** cardiovascular risk, obesity, stroke, thyroid, tsh

## Abstract

Objective

This study aimed to evaluate whether variations in normal‐range thyroid-stimulating hormone (TSH) levels affect cardiovascular outcomes in patients at high cardiovascular (CV) risk.

Methods

A cross-sectional study was conducted involving 463 middle-aged euthyroid patients (354 women, 109 men) under treatment for hypertension and dyslipidemia (TSH range: 0.5-5.2 mIU/L). Patients were grouped by TSH tertiles: lower (0.5-1.3 mIU/L), middle (>1.3-2.16 mIU/L), and upper (>2.16-5.2 mIU/L). For statistical analysis, we compared individuals with TSH ≤2.16 mIU/L (n=310) versus those with TSH >2.16 mIU/L (n=153).

Results

Anthropometric and laboratory parameters were comparable between groups, except for higher triglycerides (Tg) (151.9 ±74.9 vs. 136.0 ±72.7 mg/dL) and Tg-to-high-density lipoprotein (HDL) ratio (3.17 ±2.05 vs. 2.92 ±2.07) in those with upper-normal TSH (p<0.05). Positive correlations emerged between TSH and BMI (r=0.099, p=0.033) as well as between TSH and the Tg-to-HDL ratio (r=0.149, p=0.001). Interestingly, stroke prevalence was higher in patients whose TSH levels were in the lower-normal range (6.1% vs. 2.0%, p=0.047). Multivariate regression, adjusting for age, sex, BMI, and Tg-to-HDL ratio, showed that patients with lower-normal TSH were five times more likely to experience stroke (odds ratio (OR): 5.08; 95% confidence interval (CI): 1.15-22.25; p=0.031).

Conclusions

Within normal TSH ranges, lower-normal TSH levels were associated with a higher risk of stroke, while upper-normal TSH was linked to higher Tg levels and insulin resistance markers. These findings underscore the importance of carefully evaluating TSH levels in high-risk patients. Further prospective studies are needed to confirm these results and elucidate underlying mechanisms.

## Introduction

Thyroid hormones play a critical role in cardiovascular (CV) physiology by modulating metabolic rate, thermogenesis, body weight, heart rate, blood pressure, serum lipids, and glucose homeostasis [1]. Primary thyroid dysfunction - subclinical or overt - has been consistently tied to adverse CV outcomes and higher mortality [2-6]. Less clear, however, is whether subtle differences in thyroid-stimulating hormone (TSH) or thyroid hormone (TH) within the statistically defined “normal” range might also predispose to adverse CV outcomes. Evidence suggests that even mild variations in euthyroid individuals may carry clinical significance, especially in older adults [6-9]. An ongoing debate concerns whether current reference intervals for serum TSH should be narrowed down to account for age, ethnicity, and comorbidities. Because TSH levels trend higher with aging, the definition of “healthy thyroid function” in diverse populations is being re-examined [3].

Although TSH and free T4 reference intervals are derived statistically (2.5-97.5th percentiles), this approach does not account for differences in clinical risk. Indeed, data linking normal-range TSH variations to cardiac risk and stroke remain limited in non-thyroidal populations [9,10]. In light of this, we investigated whether normal-range TSH levels correlate with CV outcomes in a high-risk cohort already on treatment for hypertension and dyslipidemia [11].

## Materials and methods

Study population

We retrospectively analyzed 463 adult patients (354 women, 109 men), whose data were drawn from a clinical database at the Hypertension Center of Federal University of Medicine in the city of São Paulo, Brazil, from March to December 2011. The inclusion criteria were as follows: euthyroid patients (TSH: 0.5-5.2 mIU/L) without known thyroid disease or on any thyroid-related medications, who had hypertension and dyslipidemia under pharmacologic treatment. The exclusion criteria were as follows: patients who had TSH <0.5 mIU/L or >5.2 mIU/L, pregnant women, and those ≤18 years old. All participants provided written informed consent.

Clinical and laboratory evaluation

Demographic and clinical information included age, sex, CV history (coronary artery disease, stroke, peripheral artery disease), type 2 diabetes (T2DM), metabolic syndrome (MetS), medication use, smoking status, and menopausal status in women. Anthropometric data included waist circumference, weight, height, and BMI - calculated as weight (kg) / [height (m)]². Obesity was defined as BMI ≥30 kg/m².

Blood pressure was measured in the sitting position after rest. Hypertension was defined as BP ≥140/90 mmHg or ongoing antihypertensive treatment. MetS was diagnosed according to the International Diabetes Federation (IDF) or the National Cholesterol Education Programs Adult Treatment Panel III (NCEP ATP III) criteria [12,13]. Diabetes was defined as a fasting glucose level ≥126 mg/dL or any use of antidiabetic therapy.

Fasting blood samples were analyzed by enzymatic colorimetric methods for TSH, total cholesterol (TC), high-density lipoprotein (HDL) cholesterol, low-density lipoprotein (LDL) cholesterol, triglycerides (Tg), and glucose. The Friedewald formula was used to estimate LDL-C when Tg <400 mg/dL. The Tg/HDL ratio was calculated as Tg level (mg/dL)/HDL level (mg/dL) [14,15].

TSH grouping

Patients were initially divided into tertiles based on TSH: lower tertile: 0.5-1.3 mIU/L, middle tertile: >1.3-2.16 mIU/L, upper tertile: >2.16-5.2 mIU/L. For statistical comparisons, patients in the lower and middle tertiles (TSH ≤2.16 mIU/L) were combined and compared with those in the upper tertile (TSH >2.16 mIU/L), since the initial intention was to analyze and compare possible changes in behavior in the population with high TSH compared to the others.

Statistical analysis

SPSS Statistics version 22.0 (IBM Corp., Armonk, NY) was used for statistical analysis. Normal data were presented as mean ± standard deviation (SD), and non-normal data as median (interquartile range (IQR). Categorical variables were presented as frequencies and percentages. Continuous variables were compared by Student’s t-test or the Mann-Whitney U test where appropriate. Categorical variables were compared by chi-square or Fisher’s exact test. Pearson’s or Spearman’s correlation was used to assess associations. A logistic regression model (adjusted for age, sex, BMI, and Tg/HDL ratio) was employed to test TSH level associations with stroke. Statistical significance was set at p<0.05.

## Results

Baseline characteristics

Table 1 shows the demographic and clinical characteristics of the cohort. The mean TSH was 1.8 ±1.0 mIU/L and the mean age was 57.2 ±11.4 years, with 76.5% female (74.3% postmenopausal). The mean BMI was 30.94 ±6.43 kg/m²; 34.12% were overweight and 50.97% were obese. The obese population was divided into grade I (124, 26.7%), grade II (74, 15.98%), and grade III (38, 8.2%). MetS prevalence was 65.7% (IDF) and 68% (NCEP-ATPIII), and the waist circumference (WC) was 98.8 ±14.4 CM. T2DM was present in 33.5% (n=155) with a mean glucose of 110.1 ±37.4 mg/dL. A history of any major CV disease was reported by 11% (n=51), including stroke in 4.8% (n=22) and coronary disease in 8.2% (n=38). All participants were on statins and at least one antihypertensive agent, though adherence was not confirmed; they had a mean LDL of 112.5 ±29.5 mg/dL, HDL of 52 ±13.2 mg/dL, TC of 191.9 ±37.8 mg/dL, TG of 141.2 ±37.8 mg/d, and TG/HDL ratio of 3.00 ±2.05. As for BP, the mean SBP was 139.1 ±21.9 and DBP was 86.2 ±12.3 mmHg. CV disease was seen in 51 ±11% of the patients, manifesting as stroke in 4.8% (n=22) and as coronary disease in 8.2% (n=38).

**Table 1:** Clinical and laboratory characteristics of the studied population WC: waist circumference; SD: standard deviation; SBP: systolic blood pressure; DBP: diastolic blood pressure; TC: total cholesterol; HDL: high-density lipoprotein; LDL: low-density lipoprotein; TG: triglycerides; T2DM: type 2 diabetes mellitus; TSH: thyroid-stimulating hormone; BMI: body mass index; MS-NCEP: metabolic syndrome - The National Cholesterol Education Program’s Adult Treatment Panel III report; MS-IDF: metabolic syndrome - International Diabetes Federation; CVD: cardiovascular disease

Parameters	Values
Age, years, mean ±SD	57.2 ±11.4
WC, cm, mean ±SD	98.8 ±14.4
Female gender, n (%)	354 (76.5%)
Postmenopausal state, n (%)	263 (74.3%)
Heart rate, bpm, mean ±SD	73.9 ±9.4
SBP, mmHg, mean ±SD	139.1 ±21.9
DBP, mmHg, mean ±SD	86.2 ±12.3
TC, mg/dL, mean ±SD	191.9 ±37.8
HDL, mg/dL, mean ±SD	52.0 ±13.2
LDL, mg/dL, mean ±SD	112.5 ±29.5
TG, mg/dL, mean ±SD	141.2 ±73.7
TG/HDL ratio, mean ±SD	3.00 ±2.05
Glucose, mg/dL, mean ±SD	110.1 ±37.4
T2DM, n (%)	155 (33.5%)
TSH, mIU/L, mean ±SD	1.8 ±1.0
BMI, kg/m^2 ^, mean ±SD	30.94 ±6.43
Overweight, n (%)	158 (34.12%)
Obesity, n (%)	236 (50.97%)
Grade I obesity, n (%)	124 (26.7%)
Grade II obesity, n (%)	74 (15.98%)
Grade III obesity, n (%)	38 (8.2%)
MS-NCEP, n (%)	315 (68%)
MS-IDF, n (%)	304 (65.7%)
CVD, n (%)	51 (11%)
Stroke, n (%)	22 (4.8%)
Coronary disease, n (%)	38 (8.2%)

Comparisons by TSH range

When comparing TSH ≤2.16 mIU/L (n=310) versus TSH >2.16 mIU/L (n=153) (Table 2), the age (57.9 ±11.4 vs. 57.2 ±11.5 years, p=0.506), anthropometric measures [BMI (30.7 ±6.3 vs. 32.1 ±11.1 kg/m^2^, p=0.081), WC (108.0 ±12.2 vs. 108.2 ±13.6 cm, p=0.865)] and most laboratory parameters [TC (191.6 ±37.6 vs. 192.5 ±39.5 mg/dL, p=0.811), LDL (112.0 ±27.3 vs. 113.0 ±32.7 mg/dL, p=0.566), HDL (52.1 ±13.7 vs. 51.8 ±12.2 mg/dL, p=0.816), SBP (139.4 ±21.7 vs. 138.4 ±22.5 mmHg, p=0.662), DBP (86.0 ±12.0 vs. 86.7 ±12.8 mmHg, p=0.567), HR (73.7 ±9.4 vs. 74.3 ±9.4 bpm, p=0.527) and fasting glucose (109.6 ±39.6 vs. 111.3 ±32.9 mg/dL, p=0.657)] were similar. The T2DM population was also similar (32.3% vs. 35.9%, p=0.429). However, patients with TSH >2.16 mIU/L had significantly higher mean TG (136.0 ±72.7 vs. 151.9 ±74.9 mg/dL, p<0.05) and a higher Tg/HDL ratio (3.17 ±2.05 vs. 2.92 ±2.07, p<0.05) [13,14].

Cardiovascular diseases appeared more prevalent in lower normal TSH (12.9%, n=40 vs. 7.2%, n=11, p<0.05); also, stroke prevalence was higher in the same group (6.1%, n=19 vs. 2.0%, n=3, p<0.05). No significant difference was observed in terms of coronary events or other CV endpoints.

**Table 2:** Clinical and laboratory characteristics of the study population stratified by TSH parameters ^*^P<0.05 TSH (0.5-2.16 mUI/L) versus TSH (≥2.17-5.2 mUI/L), compared using Student’s t-test SD: standard deviation; TSH: thyroid-stimulating hormone; WC: waist circumference; BMI: body mass index; TC: total cholesterol; TG: triglycerides; HDL: high-density lipoprotein; LDL: low-density lipoprotein; SBP: systolic blood pressure; DBP: diastolic blood pressure; T2DM: type 2 diabetes mellitus; CVD: cardiovascular disease

Parameters	TSH 0.5-2.16 mUI/L), n=310 (67%)	TSH ≥2.17-5.2 mUI/L, n=153 (33%)	P-value
Age, years, mean ±SD	57.9 ±11.4	57.2 ±11.5	0.506
TSH, mIU/L, mean ±SD	1.28 ±0.43	3.03 ±0.76	0.000*
Glucose, mg/dL, mean ±SD	109.6 ±39.6	111.3 ±32.9	0.657
WC, cm, mean ±SD	108.0 ±12.2	108.2 ±13.6	0.865
BMI, kg/m^2 ^, mean ±SD	30.7 ±6.3	32.1 ±11.1	0.081
TC, mg/dL, mean ±SD	191.6 ±37.6	192.5 ±39.5	0.811
TG, mg/dL, mean ±SD	136.0 ±72.7	151.9 ±74.9	0.030*
HDL, mg/dL, mean ±SD	52.1 ±13.7	51.8 ±12.2	0.816
LDL, mg/dL, mean ±SD	112.0 ±27.3	113.0 ±32.7	0.566
SBP, mmHg, mean ±SD	139.4 ±21.7	138.4 ±22.5	0.662
DBP, mmHg, mean ±SD	86.0 ±12.0	86.7 ±12.8	0.567
Heart rate, bpm, mean ±SD	73.7 ±9.4	74.3 ±9.4	0.527
T2DM, (%)	32.3%	35.9%	0.429
CVD, n (%)	40 (12.9%)	11 (7.2%)	0.042*
Stroke, n (%)	19 (6.1%)	3 (2.0%)	0.034*

After adjusting for age, sex, BMI, and Tg/HDL ratio, patients with lower-normal TSH were five times more likely to have a stroke (odds ratio (OR): 5.08; 95% confidence interval (CI): 1.15-22.25; p=0.031) [15,16].

## Discussion

In this cohort of euthyroid adults at high CV risk (hypertension and dyslipidemia), our key findings were as follows: upper-normal TSH (>2.16 mIU/L) was associated with significantly higher TG and Tg/HDL ratios, suggesting heightened insulin resistance; lower-normal TSH (0.5-2.16 mIU/L) was independently associated with a fivefold increased risk of stroke, even though these patients did not display marked differences in blood pressure, fasting glucose, or lipid parameters (beyond Tg).

These findings suggest that variations in thyroid function, even within the conventionally defined 'normal' TSH range, may affect different facets of CV risk. Prior research has often associated mildly higher TSH with weight gain and adverse metabolic parameters [8,9,11-12]. Indeed, the positive correlation between TSH and BMI, as well as Tg/HDL in our study, aligns with reports suggesting subtle hypothalamic-pituitary-thyroid axis dysregulation in obesity. The proposed mechanisms include altered dopaminergic signaling, leptin-mediated modulation of thyrotropin-releasing hormone (TRH) expression, and decreased basal metabolic rate, as suggested by several authors [15,16].

Notably, although higher TSH levels were associated with adverse lipid profiles, it was the lower end of the normal TSH range that showed the strongest association with stroke in our sample. Large observational and genetic (Mendelian randomization) studies have likewise indicated that lower TSH (or higher free T4) within the euthyroid range might predispose patients to atrial fibrillation, a key stroke risk factor [17]. Similar to our findings, a study by Grassi et al. supports the notion that subclinical variations, especially in the lower-normal TSH range, may carry neurological consequences potentially unrelated to classic metabolic changes [18].

This study has certain limitations, such as its cross-sectional design and the use of only a single measurement for TSH and other laboratory parameters. Data for free T4, free T3, thyroid autoantibodies, and dietary iodine intake were not available, which limited the ability to explore mechanistic insights. Further studies with larger cohorts are needed to validate the results of this study. Additionally, future longitudinal studies with repeated measurements could provide clearer insight into how lower-normal TSH contributes to stroke risk and whether modifying thyroid function could help reduce that risk.

## Conclusions

In our cohort of euthyroid, high CV-risk patients, we observed that TSH levels in the upper-normal range (>2.16 mIU/L) were associated with elevated triglycerides and a higher TG/HDL ratio, indicating increased insulin resistance, while lower-normal TSH (0.5-2.16 mIU/L) was independently linked to a higher stroke risk. These data indicate that “normal” TSH ranges may not uniformly confer the same cardiometabolic risk profile. Larger prospective studies are needed to validate our findings and to examine whether adjusting TSH targets could improve CV outcomes in high-risk populations.
